# Deciphering the Olive Fruit Volatilome: A Multivariate Approach to Assess Cultivar Variation and Biotic Stress Response in a Changing Agroclimatic Context

**DOI:** 10.3390/plants15142243

**Published:** 2026-07-22

**Authors:** Araceli Sánchez-Ortiz, José Manuel Muñoz-Redondo, Juan Cano Rodríguez, Enrique Quesada-Moraga, José Manuel Moreno-Rojas

**Affiliations:** 1 Andalusian Institute of Agricultural and Fisheries Research and Training (IFAPA), Center Venta Del Llano, Ctra. Bailen-Motril, Km. 18.5, 23620 Mengíbar, Jaén, Spain; juan.cano.rodriguez@juntadeandalucia.es; 2 Andalusian Institute of Agricultural and Fisheries Research and Training (IFAPA), Center Alameda Del Obispo, Avda. Menéndez Pidal, SN, 14004 Córdoba, Spain; 3 Departamento de Agronomía, Unidad de Excelencia María de Maetzu, Unidad de Entomología Agrícola, Universidad de Córdoba, 14071 Córdoba, Spain; cr2qumoe@uco.es

**Keywords:** volatolomic, plant volatiles, VOCs, SPME solid-phase microextraction analysis, GC-MS, olive fruit, D-optimal response surface methodology (DoE), PLS-DA, random forest, climate change, biotic stress

## Abstract

Understanding olive tree metabolism and its interactions with biotic and abiotic factors is crucial for the sustainability and resilience of olive cultivation in a changing agroclimatic context. In response to biotic stress, plants activate complex signaling pathways that trigger the production of specialized metabolites, particularly volatile organic compounds (VOCs). This study investigates the volatolomic profile naturally emitted by whole olive fruits using an integrated metabolomic strategy that combines design of experiments (DoE), targeted and untargeted analyses, and multivariate statistics. Optimal headspace solid-phase microextraction (HS-SPME) conditions were established using 30 g of sample, a 50 °C extraction temperature, a 50 min extraction time, and a 3 min injection at 250 °C, identifying extraction time and temperature as the most critical factors influencing VOC recovery. The data demonstrated significant cultivar-dependent variation in the volatile emissions from healthy olive fruit among six representative varieties. Furthermore, robust partial least squares-discriminant analysis (PLS-DA) and random forest models provided a clear separation between healthy and damaged olive fruits, achieving high predictive accuracy (90%) and identifying key volatile biomarkers derived from the lipoxygenase (LOX) pathway. This novel multivariate optimization approach (SPME–GC/MS) represents a powerful tool for establishing a reliable chemical fingerprint of the olive fruit “volatilome” under evolving agroclimatic challenges.

## 1. Introduction

Mediterranean agricultural systems, particularly olive groves, face significant challenges arising from the evolving agroclimatic context, notably increased frequency of drought events, more extreme temperatures, and biodiversity loss. These conditions promote the spread of pests and pathogens, thereby undermining the sustainability of the system and productivity stability. Understanding olive tree metabolism and its interaction with biotic and abiotic factors is essential for addressing these challenges. In particular, biotic stressors activate complex signaling networks that induce the production of specialized metabolites, among which volatile organic compounds (VOCs) are of relevance.

Volatile metabolomics, known as “volatolomics,” is a recently introduced non-invasive approach that constitutes the study of the volatile organic compounds (VOCs) emitted by the metabolome. Plants, such as olive trees, constantly exchange information with their surrounding environment using volatile chemical signals (biogenic VOCs), including the generation of defense alarms in response to external enemies, biotic or abiotic [1].

The olive fly, *Bactrocera oleae* (Rossi), has been identified as an important threat to olive cultivation in several reports [2,3]. While fewer volatile components are detected in olive fruits than in flowers, limited data are available concerning the volatile profile of olive fruit. Nevertheless, these volatile compounds have a functional role in either attracting or repelling *Bactrocera oleae* [2,4,5,6]. Consequently, research into appropriate analytical strategies for characterizing the volatile profile of olive fruit is crucial for accurately understanding their metabolism and establishing their relationship with defense mechanisms, such as *Bactrocera oleae*’s preference for particular cultivars.

Over the years, several strategies have been developed to isolate and identify them; however, the complex and unpredictable composition of these compounds (as most of them are present at trace levels), along with the lack of standards, makes this approach difficult. The sampling technique is crucial in the development of an analytical method for the collection of volatiles emitted by fruits. Regarding plant emissions, headspace solid-phase microextraction (HS-SPME) stands out as the most commonly applied technique for volatile sampling due to its excellent sensitivity and short sampling times [7,8,9].

Nevertheless, the efficiency of volatile compound extraction using HS-SPME is mainly impacted by experimental conditions, including pH, sample quantity, ionic strength (salt addition), as well as the duration and temperature of extraction and desorption. Of these factors, the most influential ones are generally the temperature and time of extraction; thus, they are typically the focus in the majority of optimization attempts [10,11,12]. In this sense, an increase in the temperature of extraction affects the diffusion coefficient of analytes, resulting in shorter extraction times. Consequently, samples are subjected to heating to favor the release of volatile compounds into the headspace and to decrease the time required to achieve an equilibrium between the sample headspace and the stationary phase of the fused-silica fiber [7,13,14]. Thus, it can be expected to have a significant impact on the volatile extraction yields due to the interaction of these parameters. On the other hand, the optimal extraction time is linked to the partition coefficient of analytes and sample agitation. The maximum number of metabolites is extracted when equilibrium is reached. However, this might be excessively long for numerous analytes. As a result, the extraction time is typically set so that the limit of quantification is exceeded for the analyte [7].

To optimize the combination of HS-SPME factor levels that yields an optimal extraction of volatile compounds, the utilization of response surface methodology has proven to be a good approach. This methodology overcomes the limitations of traditional one-variable-at-a-time approaches, which do not consider interaction effects among factors and lead to a larger number of required experiments [13]. In this regard, a D-optimal design provides a statistically efficient approach to explore a multidimensional space, making it possible to obtain robust and accurate results with a minimal number of experimental runs [14].

After sampling, optimal extraction, and thermal desorption of volatile compounds from the SPME, the next fundamental step in their determination is analysis using an appropriate analytical platform. The most popular technique for this analysis is gas chromatography in combination with both mass spectrometry (GC-MS) and flame ionization detection (GC-FID). Notably, GC-MS is the most widely used traditional analytical technique for volatolomics. It has become a benchmark in the chemical analysis of volatile and semi-VOCs, combining characteristics that allow the qualitative identification and quantification of trace-level components present in complex sample matrices [1].

In this sense, it is important to point out that an appropriate statistical tool must be adopted to correlate chemical and biological information and correctly interpret the biological processes of plants. Several studies have aimed at examining the VOCs contributing to a plant’s scent, with a special focus on the compounds responsible for pollinator attraction [9].

The analysis of volatiles emitted by olive fruit has not yet been addressed using new metabolomic strategies, including design of experiments (DoE) and a metabolomic approach involving both targeted and untargeted identification of metabolites combined with multivariate statistical analysis. In this study, a new tool, PARAFAC2-based deconvolution and identification system for processing GC–MS data, is employed for data analysis. This system is gaining significance due to its ability to overcome many of the problems associated with targeted approaches [15]. Furthermore, a preliminary exploratory stage is followed by validation and modeling stages, which allow specific VOCs to be targeted and markers to be obtained.

The main aim of this study is to investigate and collect the largest number of volatile organic compounds (VOCs) naturally released by the whole olive fruit into the surrounding environment. Specifically, this study characterizes the overall phytochemical pattern emitted by olive fruit, with a special focus on the compounds responsible for the scent that may be involved in the attraction of insects and the characterization of varieties. This new metabolomic method provides interesting applications for characterizing the volatile profile emitted by six of the most representative olive varieties worldwide and their response to biotic factors, such as insect attacks, through a comparison of the volatiles emitted by healthy fruit and damaged fruit by simulation of biotic stress by mechanical wounding.

## 2. Results and Discussion

### 2.1. Multivariate Optimization Method by Doe for SPME-GC-TQ/MS: Optimization of the HS-SPME Conditions

In light of prior research findings and our research group’s expertise, the extraction parameters optimized for determining volatile compounds in our study were extraction temperature, extraction time, injection time, injection temperature, and sample volume. This was achieved through a D-optimal design using optimal variation ranges of the factors. In this study, the extraction temperature was varied within the range of 30 to 50 °C, a range commonly observed as optimal for characterizing volatile compounds in olive samples. This range was selected based on the findings of previous studies focusing on the analysis of volatile compounds in olive products [16,17,18]. According to these studies, a temperature range between 30 and 60 °C during the extraction phase enhances method efficiency by improving extraction kinetics, facilitating analyte diffusion, and increasing volatility without causing compound degradation. This is consistent with previous observations in berries, where temperatures above 50 °C may induce Maillard reactions and Strecker degradation, introducing non-representative variations in many compound concentrations [19]. To avoid excessively time-consuming analyses, we selected an extraction time ranging from 30 to 60 min and an injection time ranging from 3 to 10 min, which were aligned with the typical durations employed for HS-SPME-based untargeted characterization of volatile compounds [18,19,20]. The injection temperature was explored within the range of 200 to 250 °C. This temperature range was determined based on well-established protocols and prior investigations within the field [16,17,18,19,20,21]. This temperature range facilitates the desorption of volatile compounds from the SPME fiber while effectively minimizing the risk of thermal degradation or background interference. Furthermore, the sample amount required for each analysis was investigated within the bounds of 30 to 60 g, utilizing whole olives. By optimizing this parameter, we aimed to balance signal intensity with the minimization of matrix effects, thus enhancing the reliability of our results.

The summary results of the quadratic models fitted for each group of volatiles are shown in Table 1, providing valuable insights into the optimization of the HS-SPME process. All the models were highly significant (*p*-value < 0.001), displaying a non-significant lack-of-fit (*p*-value > 0.05) that indicated a good fit. The adjusted and predicted R^2^ were acceptable across all the models, showing a high percentage of variation in the responses (ranging from 0.826 to 0.958 and from 0.730 to 0.917, respectively), indicating slight differences between the two diagnostic results (indicative of the model’s robustness).

Based on the information provided in Table 1 corresponding to the factors, it was observed that time and temperature of extraction were the most influential factors in volatile extraction. These factors consistently showed significance across all families of volatile compounds (terpenes, hydrocarbons, aldehydes, and esters). Moreover, in most models, it was evident that the interactions and quadratic terms of these factors had a significant impact on volatile extraction, suggesting that they are key factors for achieving maximal extraction of the volatiles.

**Figure 1:**
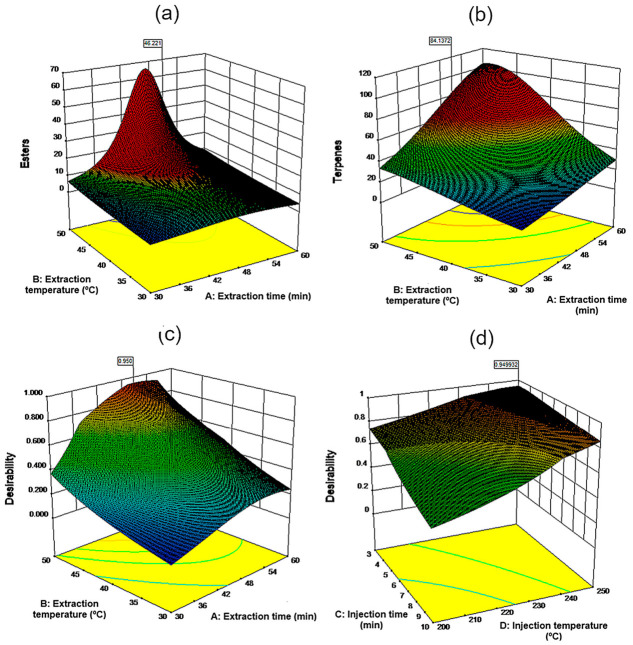
Three-dimensional (3D) surface plots of the desirability values as functions of the (**a**) time and temperature of extraction, maintaining the optimal values of sample volume and time and temperature of injection; (**b**) time and temperature of injection, maintaining the optimal values of sample volume and time and temperature of extraction. Three-dimensional (3D) surface plots of the esters (**c**) and terpenes (**d**) responses as a function of time and temperature of extraction, maintaining the optimal values of sample volume and time and temperature of injection. A: Extraction time (min), B: extraction temperature (°C), C: injection time (min), and D: injection temperature (°C). The color gradient represents the level of overall desirability, ranging from dark blue (lowest desirability, D = 0) to dark red (maximum desirability, D = 1). High-desirability regions indicate optimal operating conditions. The contour lines projected on the bottom plane represent lines of constant desirability, highlighting the transition toward the optimal region (red contours).

Figure 1a and b show how variations in these parameters impacted the final extraction of esters and terpenes, respectively, which were the families most impacted by variation in these parameters. In the case of esters, the extraction time was a critical factor. Both short and long extraction times resulted in low extraction yields, while intermediate times significantly increased the extraction yield by approximately 10-fold. The injection temperature main effect (D) was also consistently significant in all models, while the main term of injection time (C) did not consistently show a significant effect on the extraction of all the volatile families. However, its interaction with injection temperature was significant in some models, implying that while injection time did not show a standalone effect, its influence became apparent when considering this interaction.

The optimal HS-SPME experimental conditions were established through the application of a multicriteria desirability function (D). The combination of factor levels that yielded the highest global desirability function values determined the optimal HS-SPME conditions. 3D surface plots illustrating desirability values as a function of extraction temperature and extraction time (which were the most impacting factors) and temperature and time of injection, keeping the rest of the parameters at the optimized values, can be found in Figure 1c,d. The interaction between extraction time and temperature revealed an optimum at elevated temperatures (approximately 50 °C) and intermediate time levels. This effect may be attributed to the significant increase in esters, as depicted in Figure 1a. It was also noticed that extraction temperatures above 50 °C could further enhance volatile extraction yields. However, this limit was maintained to avoid potential issues related to Maillard reactions and Stecker degradation, as previously mentioned. As a result, the mathematical optimum fell on the upper limit of the tested range (50 °C) rather than inside it and was therefore regarded as a constrained optimum rather than an absolute one. Although relatively high, this temperature has also been identified as optimal for extraction in similar fruits, such as berries, where, as in our study, no degradation of the target analytes was detected. The overall impact of the interaction between time and temperature of injection is depicted in Figure 1d. Shorter injection times combined with higher temperatures led to enhanced extraction yields. However, the response range spanned by temperatures above 240 °C and times shorter than 5 min did not vary significantly. Therefore, the authors decided to maintain 3 min of extraction time and a 250 °C injection temperature as limits to ensure the complete desorption of the volatiles retained by the fiber and to prevent fiber degradation. Changes in sample mass simultaneously affected the amount of analyte and the phase ratio (β = V_headspace_/V_sample_). Although the phase ratio was not considered as a separate variable, its effect was inherently accounted for through the sample-mass factor (sample volume) included in the experimental design. Moreover, since responses were grouped by compound families with distinct volatility, this effect was implicitly considered in the optimization. Therefore, any influence of changes in headspace/sample volume on the extraction efficiency was incorporated into the desirability-based optimization. Based on the results obtained from the response surface models, the optimal conditions were attained using 30 g of sample extracted at 50 °C for 50 min, followed by a 3 min injection at 250 °C.

### 2.2. Application of the Method for the Analysis of Volatile Compounds Emitted by Olive Fruits: Pre-Processing of Chromatographic Data and Identification of Metabolites

The chromatograms of volatile compounds emitted by olive fruit in this study were pre-processed using the PARAFAC2 algorithm, a deconvolution and identification system for GC-MS data [15]. A total of 141 compounds, both identified and unidentified, were obtained, along with their LRIs and molecular characteristics, which are detailed in Supplementary Materials (Table S1).

As presented in Table S1, these compounds are grouped into four levels of identification, as defined by the Metabolomics Standards Initiative (MSI) [22]: Level 1: 29 compounds were identified at this level, meeting the criteria of matching a chemical reference standard, having an LRI within ±30 of reported literature values, and a mass spectral “match factor” (MF) of ≥850. Level 2: 10 metabolites were identified at this level, following the same criteria as Level 1 but without the use of a chemical reference standard. Level 3: 21 metabolites were identified at this level, based on an LRI within ±30 of literature data and an MF of >700. Level 4 (Unknown compounds): The remaining metabolites were grouped into this category. These unknown compounds were included in the multivariate statistical analysis approach to enhance the robustness of the results and conclusions.

Table 2 presents the results of the univariate analysis (mixed-ANOVA) for the identified volatile compounds, expressed as fold-change, across the six studied cultivars with varying tolerance levels to *Bactrocera oleae*: ‘Arbequina’, ‘Callosina’, ‘Frantoio’, ‘Hojiblanca’, ‘Nevadillo’, and ‘Picual’. The metabolites identified across both trials (Table 2 and Table 3) can be grouped into eight chemical categories: 8 aldehydes, 12 alcohols, 5 esters, 10 terpenes, 3 ketones, 18 hydrocarbons, 2 ethers, and 2 compounds classified as “others.” Of the 60 identified compounds, half (30 compounds) have previously been reported by other authors in olive fruit, virgin olive oil, or olive leaves [23,24,25,26,27,28,29].

Significant differences in volatile compounds emitted by the various cultivars are presented in Table 2. Within the aldehyde group, heptanal and hexanal showed the highest significant values for the ‘Arbequina’ cultivar, followed by ‘Frantoio’ and ‘Callosina’. Conversely, the ‘Nevadillo’ cultivar displayed the lowest levels. The ‘Arbequina’ cultivar exhibits the highest degree of resistance to *Bactrocera oleae* oviposition, while ‘Nevadillo de Jaén’ shows the highest sensitivity. Notably, hexanal has been identified in previous studies as a repellent for *Bactrocera oleae* oviposition [4,5]. ‘Frantoio’ is the cultivar that exhibited statistically significant findings regarding the alcohol group, with nonanol, 6-methyl-heptanol, and phenylethyl ethanol emitted at levels 2.5- to 3.8-fold greater than those in ‘Nevadillo’, ‘Hojiblanca’, and ‘Picual’. Nevertheless, this same cultivar, ‘Frantoio’, exhibited the lowest level of esters. Several compounds, particularly alcohols, exhibited large fold changes while remaining statistically non-significant (ns). These compounds were not specifically targeted during method optimization; therefore, their quantitative values should be considered semi-quantitative and interpreted with caution. The higher variability associated with these measurements likely contributes to the lack of statistical significance despite the large observed fold changes.

Esters (with the exception of hexyl acetate and methyl salicylate), along with aldehydes, alcohols, and ketones, are volatile compounds derived from fatty acids. They are primarily formed in plants through three pathways: lipoxygenase, α-oxidation, and β-oxidation [30]. Three ketones derived from the lipoxygenase pathway were identified in these cultivars: 1-penten-3-one, 2-pentanone, and 3-pentanone. Among these, only 2-pentanone showed significant differences, with a 5-fold higher content in ‘Nevadillo’ than in ‘Arbequina’.

Terpenoids, the largest and most diverse class of secondary metabolites, include many volatile constituents [31]. This study identified ten volatile terpenes, all previously described in other research [3,24,25,26,29,32,33], except ylangene, which was collected from the headspace of maize plants [34]. While α-pinene, farnesene, and limonene showed no significant differences between cultivars, cis-α-bergamotene and ylangene were found in significantly higher amounts in the ‘Nevadillo’ cultivar, exceeding the levels in ‘Hojiblanca’ by 132 times and ‘Arbequina’ by 154 times, respectively. Hydrocarbons were the most abundant group of volatiles emitted by olive fruits. Of these, only 1-octene and naphthalene showed statistically significant differences, with ‘Frantoio’ exhibiting the highest levels and ‘Nevadillo’ the lowest. Both 1-octene [11,35,36,37] and naphthalene [38,39] have been previously reported in virgin olive oil. Interestingly, 1-methoxyhexane, a six-carbon ether also found in virgin olive oil [11,40], was emitted by ‘Callosina’ at a concentration 101-fold greater than by ‘Frantoio’ and ‘Arbequina’.

Finally, two compounds tentatively identified as chloro-derivatives, 1-chlorooctane and 1-chloropentane, showed their highest levels in the Arbequina cultivar. Both compounds have been identified previously: 1-chlorooctane in virgin olive oil [38] and 1-chloropentane in citrus [41]. The data presented in Table 2 demonstrate that the volatile compounds emitted by healthy olive fruit vary significantly based on the cultivar. In this context, the cultivar-specific volatile differences are interpreted as reflecting the strong genetic variation among olive varieties. We do not claim a direct causal link between individual volatiles and *Bactrocera oleae* oviposition preference; rather, we propose that these varietal volatile profiles may be associated with the well-known differences in *Bactrocera oleae* susceptibility among cultivars described in scientific literature. While other agronomic and environmental factors may also influence volatile composition, the focus of this study is on the varietal component of the fruit volatile profile.

The optimized method was applied to the headspace of healthy and mechanically damaged olive fruits, and the resulting volatile compounds are summarized in Table 3. To isolate the effect of damage independently of sampling time, the data were normalized using a multi-level decomposition approach, ensuring that the observed differences could be attributed only to fruit damage. Of the 60 volatile compounds identified in the study, only 14 showed statistically significant differences between mechanically damaged and healthy olive fruit.

Aldehydes, alcohols, and ketones are derivatives of the lipoxygenase (LOX) pathway. Specifically, E-2-hexenal, Z-3-hexenol, 1-penten-3-one, and 3-pentanone were emitted in higher amounts from damaged fruit. According to [42], the primary products of the LOX pathway, also known as LOX derivatives, are C6 aldehydes. Z-3-hexenal, for instance, is quickly converted to the more stable E-2-hexenal, which was emitted at levels up to seven times higher in damaged fruits. Recent evidence identifies ROS and RNS as upstream regulators of VOC biosynthesis upon damage, linking oxidative signaling to defense metabolite production; thus, volatile emissions reflect redox-controlled stress responses [43].

Terpenes represent an important family of secondary metabolites emitted by plants, both spontaneously and under stress (biotic or abiotic) conditions. In damaged olive fruit, only α-pinene showed significant differences, with nearly triple the quantity compared to undamaged olive fruit. Kokkari et al. [25] also detected α-pinene in greater amounts in olive fruit of cv. Megaritiki, irrespective of the fruit’s growth stage.

The pronounced induction of LOX-pathway-derived volatiles following mechanical wounding is widely regarded as a rapid, non-specific response associated with membrane disruption and subsequent activation of the lipoxygenase pathway. Accordingly, within our experimental framework, these compounds are primarily interpreted as by-products of generic tissue damage rather than as indicators of herbivore-specific defensive signaling. Indeed, although fine-needle puncture was used as a controlled proxy for oviposition because it reproduces the initial physical injury and activates wound-related pathways, including those linked to LOX-derived volatiles, this model does not fully reproduce the biochemical complexity of *Bactrocera oleae* oviposition. Therefore, these results provide a robust description of the cultivar-dependent wound response under controlled conditions, while future studies using real oviposition will be needed to assess additional insect-derived effects on the volatile profile.

### 2.3. Multivariate Approach: Volatile Compound Selection and Modeling for Damaged Fruit and Cultivar Differentiation

**Figure 2:**
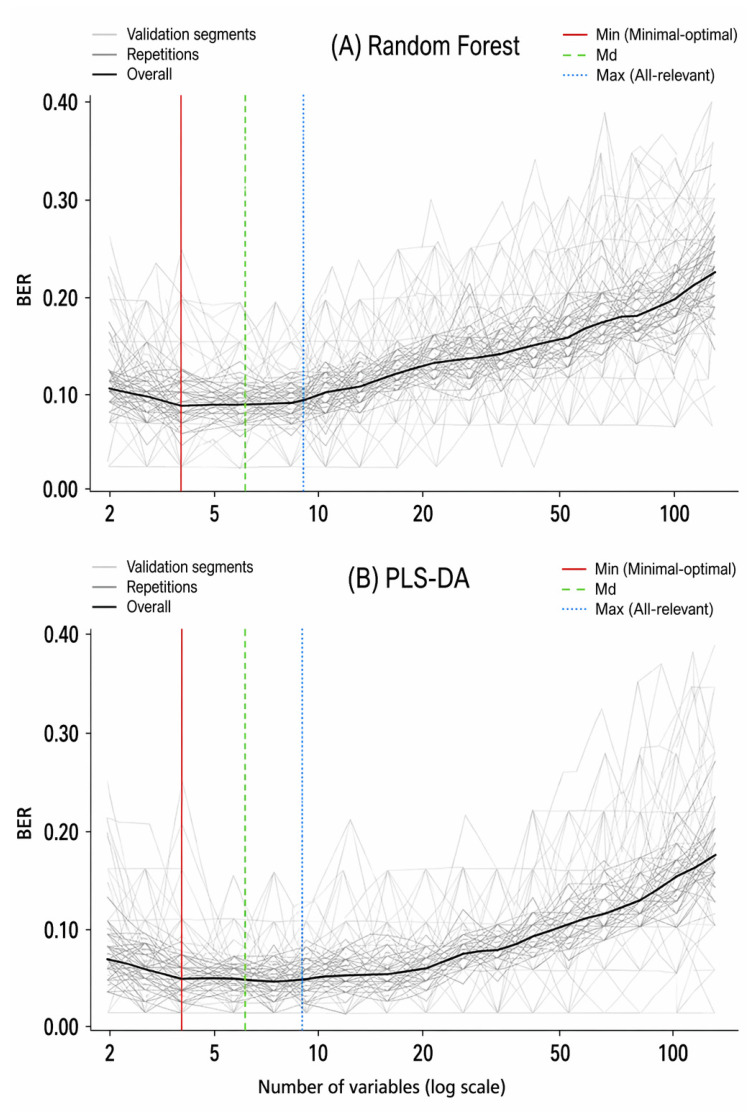
Validation curves for random forest (**A**) and PLS-DA (**B**) models used for the classification of olive fruits from six cultivars. On the left, thin lines represent individual validation segments, medium-thickness lines correspond to validation repetitions, and the thick line represents the mean validation performance. On the right, the Min (minimal-optimal, light green), Md (dark green), and Max (all-relevant, blue) variable subsets are indicated.

A multivariate approach was used, focusing on volatile compound selection and subsequent modeling to differentiate between damaged fruit and various cultivars. We applied a multi-level decomposition to the data to reduce the influence of the sampling time factor, allowing us to primarily assess the effect of fruit damage. Next, two classification models were built to select volatile markers related to “damaged fruit.” Both random forest (RF) and partial least squares-discriminant analysis (PLS-DA) models, using all volatile metabolites, were optimized and validated to identify the most discriminative compounds (Figure 2). These selected compounds were then used to optimize and validate a new model.

**Figure 3:**
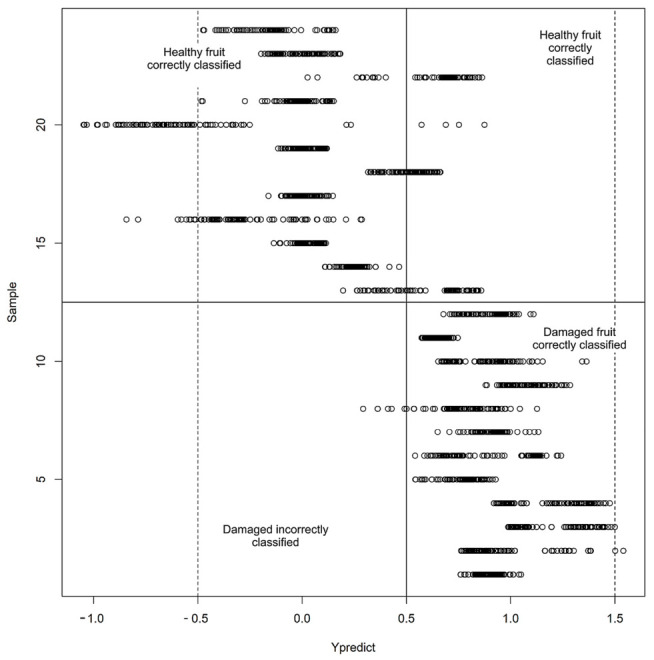
Y-prediction plot of the PLS-DA models developed using the volatile compounds emitted by olive fruits for the discrimination between healthy and damaged samples. The upper region of the plot contains healthy olive fruits correctly classified as “healthy fruit”, whereas the lower right region shows damaged olive fruits correctly classified as “damaged fruit”. The lower left region corresponds to healthy samples misclassified as “damaged fruit” (false positives).

In total, eleven compounds were selected by both validated models. Other identified compounds did not show significant changes due to fruit damage, so they were not considered as markers. Finally, these markers were used to refine a PLS-DA model (Table 4 and Figure 3). The final model, built with the most discriminative compounds, achieved an overall mean balanced error rate (BER) of 0.10, indicating a high hit rate of 90%. All damaged samples were correctly classified as “damaged fruit.” However, unexpectedly, “healthy fruit samples” were misclassified by the model as “damaged fruit samples,” though with a low mean class error of 0.19. The “false positives” for damaged fruit might be related to damage incurred during the handling of the olive fruit samples. It should be noted, however, that no visible signs of damage were detected during fruit handling and processing. Therefore, although handling-related damage cannot be completely ruled out, another plausible hypothesis is that certain volatile compounds are emitted by both healthy and damaged fruits, leading to highly similar volatile profiles and potentially contributing to the occurrence of false positives.

**Figure 4:**
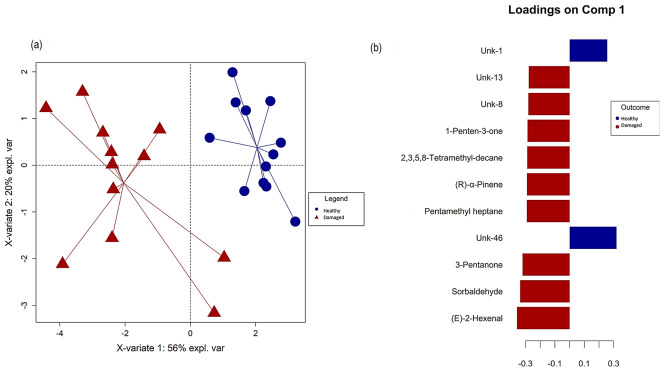
Scores (**a**) and loadings (**b**) plots obtained from the PLS-DA model based on the selected volatile compounds emitted by healthy (HF) and damaged (DF) olive fruits. The scores plot (**a**) shows the distribution of samples and their separation according to fruit health status, while the loadings plot (**b**) illustrates the contribution of the selected volatile compounds to the discrimination between HF and DF samples.

Regarding volatile compound markers for fruit health and cultivar differentiation, partial least squares discriminant analysis (PLS-DA) was performed to identify volatile compounds serving as markers for fruit health and insect damage, specifically due to oviposition (Figure 4). Following model optimization, several compounds showed increased concentrations in damaged fruit: two aldehydes (E-2-hexenal and 2,4-hexadienal, also known as “sorbaldehyde”), a terpene (α-pinene), two ketones (3-pentanone and 1-penten-3-one), and two aromatic hydrocarbons (2,3,5,8-tetramethyldecane and 2,2,4,6,6-pentamethylheptane). E-2-hexenal exhibited the most significant concentration increase, as supported by ANOVA results (Table 3). This C6 aldehyde and the C5 carbonyls (3-pentanone and 1-pente-3-one) are direct products of the lipoxygenase metabolic pathway, formed by fatty acid degradation when tissue integrity is compromised [44].

In agreement with previous observations [45], the multivariate analysis effectively differentiates cultivars and damage conditions based on bulk volatile profiling; this approach does not allow the resolution of localized, cell-type-specific responses at the wound site. Therefore, although the method provides robust insight into systemic volatile changes, future studies incorporating spatially resolved analyses are needed to better interpret the lipoxygenase (LOX) pathway signature and elucidate the mechanisms underlying olive defense against insect attack.

In relation to cultivar-specific volatile profiles, a multivariate approach was also employed to analyze differences in volatile profiles attributable to cultivar variations. Figure S1 illustrates the scores and loading plots from a PLS-DA performed on volatile compounds emitted by several cultivars. For this analysis, a two-level factor model was fitted to decompose variability along the factorial design directions. Variable selection was conducted using an iterative method, resulting in a new model that achieved a 100% successful classification rate. Cultivars such as ‘Arbequina’ and ‘Picual’ were clearly differentiated.

## 3. Materials and Methods

### 3.1. Sampling Process and Sample Preparation of Olive Fruit: Simulation of Biotic Stress by Mechanical Wounding

Olive fruits were collected from the experimental olive grove at the IFAPA Center Venta del Llano, Mengíbar (Jaén), Spain. Three olive trees were selected to obtain fruit for different purposes. An initial batch of olives (2 kg of the ‘Picual’ cultivar) was harvested in late September for optimization of the volatile compound analysis method. A second batch was harvested in mid-November 2020 to prepare quality control samples (1 kg of ‘Picual’ fruit) and to apply the optimized method. For the latter, 1 kg of fruit was collected from six cultivars selected for their differing tolerance to the insect Bactrocera oleae: ‘Arbequina’, ‘Frantoio’, ‘Callosina’, ‘Picual’, ‘Hojiblanca’, and ‘Nevadillo de Jaén’. Finally, a third batch including all six varieties was collected under the same conditions in mid-December 2020. According to Quesada-Moraga et al. [46], the ‘Arbequina’ cultivar exhibits the highest resistance to *Bactrocera oleae* oviposition, whereas ‘Nevadillo de Jaén’ shows the greatest susceptibility.

The optimized method was applied to characterize the volatile profile emitted by the six selected cultivars and to assess changes in fruit volatiles after mechanically induced damage mimicking the physical injury caused by insects. Oviposition by *Bactrocera oleae*, which produces a puncture in the olive during oviposition, was used as the biological model for this type of damage. Mechanical needle puncture was selected as a controlled proxy because it activates wound-induced volatile pathways in olive fruit and allows the extent and timing of damage to be standardized across cultivars. This approach enables the specific evaluation of tissue injury effects on the fruit volatile profile, independently of additional factors such as enzymes or other compounds released by the insect during real oviposition [47,48].

Finally, for each selected variety, fruits subjected to puncture were classified as “damaged fruit”, whereas the unpunctured batch was classified as “healthy fruit”. For all experiments, fruits were stored at room temperature (22–25 °C) for up to 72 h. Samples were prepared in triplicate from 30 g of fruit.

### 3.2. Reagents and Materials

SPME (solid-phase microextraction) fibers coated with 50/30 µm DVB/CAR/PDMS, a blend of n-alkane standards containing 8 to 20 carbon atoms (40 mg/L each, in hexane), and chromatography-grade volatile compound standards with a purities higher than 95% were provided by Supelco (Bellefonte, PA, USA): (E)-2-hexenal, (E,E)-2,4-heptadienal, 2,4-hexadienal, (R)-α-pinene, (Z)-hex-3-en-1-ol, 1,2,3-trimethylbenzene, 1-hexanol, 1-nonanol, 1-pentanol, 1-penten-3-one, 2-pentanone, 3-hexenol acetate, 3-pentanone, benzaldehyde, benzyl alcohol, copaene, cyclohexane, decane, ethanol, ethylbenzene, farnesane, heptanal, heptane, hexanal, hexyl acetate, limonene, nonanal, octanal, o-xylene, toluene, undecane, 3,5-dimethyloctane, caryophyllene, cis-α-bergamotene, methyl benzoate, methyl salicylate, pentadecane, phenylethyl alcohol, propylbenzene, tetradecane, trans-α-bergamotene.

### 3.3. Optimization of the Extraction Method of Volatile Compounds Emitted by Olive Fruit: “HS-SPME-GC-MS” by DoE

HS-SPME was used to optimize the extraction of volatile compounds from the olive samples; a “D-optimal” response surface methodology was employed. The factors under investigation included the extraction time (varied between 30 and 60 min), extraction temperature (30 to 50 °C), injection time (3 to 10 min), injection temperature (200 to 250 °C), and sample volume (30 to 60 g). Changes in sample mass simultaneously affected the amount of analyte and the phase ratio (β = Vheadspace/Vsample). Although the phase ratio was not considered as a separate variable, its effect was inherently accounted for through the sample-mass factor (sample volume) included in the experimental design. Moreover, since responses were grouped by compound families with distinct volatility, this effect was implicitly considered in the optimization. Therefore, any influence of changes in headspace/sample volume on the extraction efficiency was incorporated into the desirability-based optimization. Fruits were introduced into a Pyrex glass bottle with a 250 mL capacity and a screw cap with a silicone septum, through which the fiber was introduced and exposed to the headspace of the sample. The HS-SPME fiber used was coated with DVB/CAR/PDMS, which was selected for its broader polarity affinity, enabling the extraction of a greater variety of metabolites in samples with heterogeneous metabolite compositions at trace levels. The D-optimal approach was selected for its ability to efficiently explore the multidimensional parameter space and identify the optimal combination of these variables to maximize the extraction efficiency. In addition, this experimental design allowed a systematic and comprehensive examination of the extraction while keeping a low number of runs (required experiments), leading to more robust and reliable outcomes [49]. Subsequently, a quadratic linear model was fitted to the data obtained from the experimental conditions using stepwise regression (forward and backward selection), optimized by the Bayesian information criterion (BIC) for coefficient selection. Consequently, the following mathematical formula was derived for each model:

$$Y_{i}=\;\alpha_{0}+\;\sum_{i=1}^{5}\beta_{i}X_{i}+\;\sum_{i=1}^{5}\beta_{ii}X_{i}^{2}+\;\sum_{i<j}^{5}\beta_{ij}X_{i}X_{j}$$

where *Y_i_* is the predicted response (summation of the peak areas), *α*_0_ is the intercept, *β_i_*, *β_ii_*_,_ and *β_ij_* are the coefficients indicating the effect on the responses, and *X_i_* and *X_j_* are the factors.

Four main families of compounds (terpenes, hydrocarbons, aldehydes, and esters) were used as responses, obtaining a quadratic model for each group. The models were assessed based on the coefficient of determination (both adjusted and predicted R^2^), significance of the model and model coefficients (calculated from the F distribution and considered significant at a *p*-value ≤ 0.05), and the lack of fit (considered non-significant at a *p*-value > 0.05). To reduce the complexity of the models, non-significant terms were removed for making predictions.

To determine the extraction conditions that achieved the best balance in extracting the four volatile compound families, a desirability function (D) proposed by Derringer and Suich (Derringer & Suich, 1980) [50] was employed. This consisted of transforming the individual responses of each family of compounds from their original experimental values into individual desirability functions, which ranged from 0 to 1 and represented unacceptable to fully desirable response levels, respectively. Subsequently, a global desirability function was obtained by calculating the geometric mean of these individual desirability values.

Finally, experimental responses were obtained under the optimized extraction conditions to validate the optimization methodology by comparing them with the theoretical values obtained from the response surface models. Model fitting and optimization were performed with the Design-Expert v.10 software.

### 3.4. Chromatographic and Mass Spectrometry Analysis: Data Preprocessing, Metabolite Identification, Variable Selection, and Modeling

Volatile compounds extracted by the SPME fiber were desorbed into the GC-MS injector in splitless mode, and the volatile compounds were separated using a Supelcowax 10 fused-silica capillary column (60 m × 0.25 mm × 0.25 µm film thickness; (Supelco, Bellefonte, PA, USA). A Bruker 456-GC/TQ Scion chromatographic instrument (Bruker/SCION Instruments, Goes, The Netherlands) equipped with an autosampler was used, with parameters set according to Sánchez-Ortiz [51]. Helium was used as the carrier gas at a flow rate of 1.5 mL/min. The oven temperature program was as follows: an initial hold at 40 °C for 10 min, followed by a ramp at 3 °C/min to a final temperature of 200 °C. A cleaning step was then added, involving a ramp at 20 °C/min to 250 °C, held for 5 min. The transfer line and ion source temperatures were set at 275 °C and 200 °C, respectively. Electron ionization (EI) mode with positive polarity was selected, and data were acquired in full-scan mode, ranging from 30 to 400 m/z.

GC-MS data were processed using PARADISe version 3.9 (PARAFAC2), with metabolite identification performed against the NIST version 17 library [15]. This software has been successfully applied to VOC data to assess the quality and authenticity of various foods. The identification of volatile compounds (Table S1 in Supplementary Materials) was conducted according to the Metabolomics Standards Initiative (MSI) guidelines, employing a four-level system [22]: (1) identified compounds, (2) putatively annotated compounds, (3) putatively characterized compound classes, and (4) unknown compounds. A semi-quantitative method was used, reporting each metabolite’s instrument response relative to that of another metabolite with the lowest level (fold-change ratio).

### 3.5. Statistical Analysis

Data collected on olive fruit volatile compounds using the developed methodology were then submitted to statistical analysis. To isolate the effect of both cultivar and insect damage on olive fruit, irrespective of the sampling time, a multi-level decomposition (or normalization) was applied to the data. This analysis ensures that observed differences are attributable solely to biological factors: cultivar and olive fruit damage. First, a univariate statistical analysis was performed using R v.3.5.0. A two-way mixed-variance analysis of variance (ANOVA) was performed, considering both cultivar and bite insect as factors. A Tukey HSD post-hoc test was applied to the dataset to identify statistically significant differences at *p* < 0.05. Results were expressed semi-quantitatively as fold-change, calculated as the ratio between the value of a volatile compound in a sample and the smallest value within its respective group (either cultivar or healthy/damaged fruit).

Following this, a preliminary exploratory analysis of the data was performed using principal component analysis (PCA). For classification tasks and to select the most discriminative compounds, partial least squares discriminant analysis (PLS-DA) and random forest (RF) models were employed. Model optimization was based on a double cross-validation scheme, consistent with the methodology described by Muñoz-Redondo et al. [52]. Random forest models were built within the MUVR framework in R [53] using the default settings, with 150 trees for the inner cross-validation loop and 300 trees for the outer (consensus) loop. The number of predictors sampled at each split was determined by the square root of the number of predictors for classification, with an upper limit of 150. For PLS-DA, the number of components was not fixed in advance but optimized internally by MUVR through the same nested cross-validation procedure used for variable selection, within a maximum of 5 components.

## 4. Conclusions

A multivariate optimization methodology based on design of experiments (DoE) was implemented, utilizing the results generated by the response surface models. The optimal HS-SPME conditions for volatile compounds emitted by olive fruit were determined using this approach: 30 g of sample extracted at 50 °C for 50 min, followed by a 3 min injection at 250 °C. Time and extraction were the most influential factors in volatile extraction. The data presented in this study demonstrate significant cultivar-dependent variation in the volatile compounds emitted by healthy olive fruit. Among all volatile compounds identified in healthy and damaged olive fruit, aldehydes, alcohols, and ketones derived from the lipoxygenase (LOX) pathway were emitted at significantly higher levels in damaged fruit, notably E-2-hexenal, Z-3-hexenol, 1-penten-3-one, and 3-pentanone. The Y-prediction plots of the PLS-DA models demonstrated a clear separation between healthy and damaged olive fruits, indicating good predictive accuracy. Most samples were correctly classified, confirming the robustness of the models. These results suggest that the volatile compounds emitted by the olive fruits provide reliable markers for discriminating between healthy and damaged samples when biotic stress is simulated through mechanical wounding.

The optimization of a solid-phase microextraction–gas chromatography/mass spectrometry (SPME–GC/MS) method provides an effective approach for the comprehensive characterization of the olive “volatilome”. The combined application of targeted and untargeted metabolomic strategies, supported by design of experiments (DoE) and multivariate statistical analysis, enables improved metabolite coverage, enhanced analytical robustness, and reliable identification of biomarkers associated with the evaluation of biotic and abiotic factors. However, the fruit damage was experimentally simulated rather than caused by real oviposition, so the findings should be interpreted as a controlled approximation of the natural interaction. These findings provide a useful framework for future studies aimed at comparing mechanically induced and insect-induced volatile responses.

## Figures and Tables

**Table 1:** Summary results of the quadratic models fitted for each family of volatiles during the HS-SPME optimization.

Family of Volatiles	Lack of Fit (*p*-Value)	R^2^_Adj_^a^	R^2^_Pred_^b^	Model (*p*-Value)	Significant Coefficients ^c^
Terpenes	0.252	0.932	0.832	<0.001	A, B, C, D, AB, AC, AD, BE, B^2^
Hydrocarbons	0.187	0.958	0.917	<0.001	A, B, D, E, AB, BC, CD, A^2^
Aldehydes	0.711	0.901	0.819	<0.001	A, B, D, E, AB, BC, A^2^
Esters	0.934	0.826	0.730	<0.001	A, B, D, E, AB, BC, CD, A^2^, E^2^

^a^ Adjusted coefficient of determination. ^b^ Predicted coefficient of determination. ^c^ Significant coefficients of each model at a *p*-value ≤ 0.05, providing insights into the influence of individual, interaction, and quadratic terms of each factor. A: extraction time; B: extraction temperature; C: injection time; D: injection temperature; E: sample amount.

**Table 2:** Two-way mixed analysis of variance (ANOVA) of volatile compounds emitted by olive fruit from the cultivars Arbequina (A), Callosina (C), Frantoio (F), Hojiblanca (H), Nevadillo (N) and Picual (P), expressed as fold changes.

Compound	A	C	F	H	N	P	*p*-Value
Aldehydes							
(E)-2-Hexenal	1.0	1.6	1.1	2.0	1.6	1.5	ns
(E,E)-2,4-Heptadienal	2.2	1.8	2.0	1.0	1.9	1.3	ns
Benzaldehyde	1.2	1.3	1.1	1.4	1.0	1.2	ns
Heptanal	4.0 a	1.7 b	1.9 b	1.5 b	1.0 b	1.5 b	***
Hexanal	3.3 a	2.0 ab	2.8 ab	1.0 b	1.0 b	1.0 b	**
Nonanal	1.8 a	1.4 ab	1.9 a	1.6 ab	1.0 b	1.6 ab	**
Octanal	1.5	1.3	1.5	1.5	1.0	1.4	ns
2,4-Hexadienal	1.0	1.7	1.3	1.9	1.5	1.4	ns
Alcohols							
(Z)-Hex-3-en-1-ol	1.5	4.1	1.0	2.2	2.0	3.1	ns
1-Hexanol	2.2	3.5	3.4	1.0	1.5	1.4	ns
1-Nonanol	2.4 ab	1.9 ab	3.8 a	1.0 b	1.2 b	2.6 ab	**
1-Pentanol	1.9	1.7	2.2	1.0	1.9	2.3	ns
2-Ethyl-1-hexanol	1.3	1.3	1.2	1.1	1.0	1.2	ns
2-Propylheptanol	1.1	1.2	1.1	1.2	1.0	1.2	ns
6-Methyl-1-heptanol	2.5 ab	1.7 bc	2.7 a	1.2 c	1.0 c	1.9 abc	***
Benzyl Alcohol	1.1	1.1	1.0	1.0	1.0	1.1	ns
Butyl dioxitol	1.4	1.5	1.0	2.1	1.6	1.5	ns
Ethanol	1.2	5.0	52.4	1.0	10.4	4.1	ns
Phenylethyl Alcohol	1.2 b	1.2 b	2.5 a	1.0 b	1.8 ab	1.0 b	***
β-Butoxyethanol	1.1	1.1	1.3	1.0	1.0	1.2	ns
Esters							
1-Methoxy-2-propyl acetate	1.2	1.4	1.2	1.0	1.0	1.4	ns
3-Hexenol acetate	4.1 c	80.2 a	1.0 c	12.0 bc	67.8 ab	32.6 abc	**
Hexyl acetate	1.3 b	13.0 ab	1.0 b	3.0 ab	15.1 a	4.4 ab	*
Methyl benzoate	19.5 a	4.2 a	1.0 a	1.9 a	9.1 a	24.5 a	*
Methyl salicylate	1.4 b	1.9 b	1.0 b	2.0 b	13.2 a	4.0 ab	**
Terpenes							
α-Pinene	1.0	1.6	1.7	1.9	1.1	2.0	ns
Caryophyllene	1.6 b	8.3 ab	1.0 b	5.5 b	18.4 a	1.8 b	***
cis-α-Bergamotene	64.7 bc	77.9 b	6.8 d	1.0 d	31.5 cd	132.2 a	***
Copaene	1.1 b	17.5 ab	1.0 b	13.8 b	33.9 a	1.1 b	***
Farnesane	1.5	1.3	1.5	1.0	1.2	1.3	ns
Limonene	1.9	1.0	1.8	1.5	2.0	1.8	ns
Trans-α-bergamotene	15.1 a	7.0 b	1.9 bc	1.0 c	1.5 c	7.0 b	***
Ylangene	1.0 b	82.4 ab	1.2 b	97.8 a	154.4 a	2.3 b	***
α-Cubebene	1.0 b	10.8 ab	1.1 b	7.1 b	24.8 a	5.0 b	***
δ-Cadinene	1.1 b	18.3 ab	1.0 b	10.7 ab	42.9 a	1.4 b	**
Ketones							
1-Penten-3-one	1.0	1.6	1.5	2.8	1.7	1.8	ns
2-Pentanone	1.0 c	4.0 ab	1.7 bc	2.5 bc	5.3 a	3.0 abc	***
3-Pentanone	1.1	2.2	1.0	1.4	1.5	1.6	ns
Hydrocarbons							
1-Octene	2.8 ab	1.7 bc	3.0 a	1.4 c	1.0 c	2.0 abc	***
2,2,4,6,6-Pentamethylheptane	1.4	2.2	1.9	2.2	1.0	2.4	ns
2,3,5,8-Tetramethyl-decane	1.0	1.5	1.4	1.4	1.2	1.5	ns
3,5-Dimethyloctane	1.0	1.8	1.4	1.8	2.3	1.8	ns
4-Methyldecane	1.0	1.3	1.0	1.2	1.0	1.3	ns
Cyclohexane	2.0	1.5	2.3	3.2	1.0	4.6	ns
Decane	1.0	1.3	1.1	1.3	1.2	1.4	ns
Ethylbenzene	1.0	1.2	1.2	1.3	1.1	1.4	ns
Heptane	1.1	1.6	3.6	3.1	1.0	1.5	ns
o-Xylene	1.1	1.1	1.1	1.3	1.0	1.2	ns
Pentadecane	1.6	1.6	1.9	1.0	1.3	2.0	ns
Propylbenzene	1.1	1.1	1.1	1.1	1.0	1.2	ns
Tetradecane	1.6	1.6	1.9	1.0	1.3	1.5	ns
Toluene	1.0	1.1	1.0	1.1	1.0	1.2	ns
Tridecane	1.4	1.4	1.4	1.0	1.2	1.3	ns
Undecane	1.0	1.2	1.0	1.2	1.1	1.2	ns
1,2,3-Trimethylbenzene	1.0	1.1	1.1	1.0	1.0	1.2	ns
Naphthalene	1.3 b	4.0 ab	1.9 ab	2.5 ab	9.3 a	1.0 b	*
Ethers							
1-Methoxyhexane	1.0 b	101.0 a	1.7 b	70.5 ab	53.4 ab	40.9 ab	*
Dipropylene glycol monomethyl ether	1.3	1.2	1.0	2.7	1.9	1.3	ns
Others							
1-Chlorooctane	2.6 a	1.8 abc	2.6 a	1.3 bc	1.0 c	2.2 ab	**
1-Chloropentane	5.8 a	2.3 bc	4.7 ab	1.0 c	1.2 c	1.4 c	***

Different letters within each row indicate significant differences according to Tukey’s test (ns = not significant, * *p* < 0.05, ** *p* < 0.01 and *** *p* < 0.001).

**Table 3:** Two-way mixed analysis of variance (ANOVA) of volatile compounds identified for healthy and damaged olive fruit, expressed as fold changes.

Compound	Healthy	Damaged	*p*-Value
Aldehydes			
(E)-2-Hexenal	1.0 b	7.0 a	***
(E,E)-2,4-Heptadienal	1.2	1.0	ns
Benzaldehyde	1.0	1.0	ns
Heptanal	1.0	1.0	ns
Hexanal	1.0	1.3	ns
Nonanal	1.0	1.1	ns
Octanal	1.0	1.0	ns
Sorbaldehyde (2,4-Hexadienal)	1.0 b	3.1 a	**
Alcohols			
(Z)-Hex-3-en-1-ol	1.0 b	2.0 a	*
1-Hexanol	1.0	1.4	ns
1-Nonanol	1.0	1.1	ns
1-Pentanol	1.0 b	1.6 a	*
2-Ethyl-1-hexanol	1.0	1.1	ns
2-Propylheptanol	1.0 b	1.3 a	*
6-Methyl-1-heptanol	1.0	1.0	ns
Benzyl Alcohol	1.0	1.0	ns
Butyl dioxitol	1.4	1.0	ns
Ethanol	1.0	1.4	ns
Phenylethyl Alcohol	1.0	1.2	ns
β-Butoxyethanol	1.1	1.0	ns
Esters			
1-Methoxy-2-propyl acetate	1.0	1.0	ns
3-Hexenol acetate	1.0	1.3	ns
Hexyl acetate	1.0	1.2	ns
Methyl benzoate	1.0	1.3	ns
Methyl salicylate	1.0	1.0	ns
Terpenes			
α-Pinene	1.0 b	2.8 a	**
Caryophyllene	1.0	1.1	ns
cis-α-Bergamotene	1.0	1.1	ns
Copaene	1.0	1.1	ns
Farnesane	1.1	1.0	ns
Limonene	1.3	1.0	ns
trans-α-Bergamotene	1.0	1.1	ns
Ylangene	1.0	1.1	ns
α-Cubebene	1.0	1.0	ns
δ-Cadinene	1.0	1.1	ns
Ketones			
1-Penten-3-one	1.0 b	4.3 a	**
2-Pentanone	1.2	1.0	ns
3-Pentanone	1.0 b	2.4 a	**
Hydrocarbons			
1-Octene	1.0	1.0	ns
2,2,4,6,6-Pentamethylheptane	1.0 b	2.8 a	**
2,3,5,8-Tetramethyl-decane	1.0 b	1.8 a	**
3,5-Dimethyloctane	1.0 b	1.8 a	*
4-Methyldecane	1.0 b	1.5 a	*
Cyclohexane	1.0	1.8	ns
Decane	1.0 b	1.5 a	*
Ethylbenzene	1.0	1.2	ns
Heptane	1.0	1.1	ns
o-Xylene	1.0	1.1	ns
Pentadecane	1.0	1.0	ns
Propylbenzene	1.0 b	1.2 a	**
Tetradecane	1.0	1.0	ns
Toluene	1.0	1.1	ns
Tridecane	1.0	1.0	ns
Undecane	1.0 b	1.2 a	*
1,2,3-Trimethylbenzene	1.0 b	1.1 a	*
Naphthalene (aromatic)	1.0	1.1	ns
Ethers			
1-Methoxyhexane	1.0	1.1	ns
Dipropylene glycol monomethyl ether	1.2	1.0	ns
Others			
1-Chlorooctane	1.0	1.0	ns
1-Chloropentane	1.2	1.0	ns

Different letters in different columns indicate significant differences according to Tukey’s test (ns = not significant, * *p* < 0.05, ** *p* < 0.01 and *** *p* < 0.001).

**Table 4:** Performance or validation table of the PLS-DA models carried out for healthy and damaged olive fruits.

Classification Group ^a^	Mean Overall BER ^b^	Class	Mean Class Error BER ^c^
Healthy	0.10 ± 0.03	Healthy	0.00 ± 0.02
Damaged		Damaged	0.19 ± 0.05

^a^ PLS-DA models constructed using either all variables or variables selected through an iterative procedure combining PLS-DA and random forest (variable importance in projection/VIP). ^b^ Mean overall BER values with the standard deviation calculated based on PLS-DA submodels in a double-cross validation scheme. ^c^ Mean class BER values with the standard deviation calculated based on PLS-DA submodels and random forest.

## Data Availability

Data are available in the paper and its Supplementary Information Files.

## Supplementary Materials

The following supporting information can be downloaded at https://www.mdpi.com/article/10.3390/plants15142243/s1, Figure S1. Scores (a) and loadings (b) plots obtained from the partial least squares discriminant analysis (PLS-DA) based on the selected volatile compounds for olive cultivar discrimination. The scores plot (a) shows the distribution and clustering of samples according to cultivar, highlighting the separation achieved by the model. The loadings plot (b) identifies the volatile compounds that contribute most strongly to cultivar differentiation; Table S1. Metabolite identification and classification according to the Metabolomics Standards Initiative (MSI). Metabolites are grouped into four identification levels (Levels 1–4, with Level 4 indicating unknown compounds) and listed by retention time (min), along with their corresponding CAS number, reverse match factor (RMF), match factor (MF), experimental linear retention index (LRIexp), and theoretical linear retention index (LRIt).

## Abbreviations

The following abbreviations are used in this manuscript:

DoE	Design of Experiments
SPME	Solid-Phase Microextraction
GC-MS	Gas Chromatography coupled to Mass Spectrometry
VOC	Volatile Organic Compound
PLS-DA	Partial Least Squares Discriminant Analysis
