# Supplementary material for: Deciphering the Olive Fruit Volatilome: A Multivariate Approach to Assess Cultivar Variation and Biotic Stress Response in a Changing Agroclimatic Context
Source: Plants (Basel). 2026 Jul 22;15(14):2243. doi: 10.3390/plants15142243 (PMC13417002; doi:10.3390/plants15142243)
Supplement: Supplementary file 1 [file plants-15-02243-s001.zip › plants-4424238-supplementary/Table S1.pdf]

Table S1. Metabolite identification and classification according to the Metabolomics Standards Initiative (MSI). Metabolites are grouped into four identification levels (Level 1- 4, with Level 4 indicating unknown compounds) and listed by retention time (min), along with their corresponding CAS number, reverse match factor (RMF), match factor (MF), experimental linear retention index (LRI_{exp}), and theoretical linear retention index (LRI_t).

N	Rt (min)	Metabolite	LRI exp	LRI t	ID Level	CAS	RMF	MF
1	6.13	Unk-1			4			
2	7.54	Heptane	738	700	1	142-82-5	910	889
3	7.88	Cyclohexane	752	752	1	110-82-7	950	948
4	9.01	Unk-2			4			
5	9.52	Unk-3			4			
6	10.01	1-Octene	840	830	2	111-66-0	890	826
7	10.02	Unk-4	840		4		957	954
8	12.78	Ethanol	942	934	1	64-17-5	915	915
9	13.02	Amyl chloride	949	945	2	543-59-9	894	726
10	13.18	Unk-5	955		4		920	883
11	13.22	Hexyl methyl ether	956	941	2	4747-07-3	924	920
12	13.32	2,2,4,6,6-Pentamethylheptane	959	957	2	13475-82-6	937	929
13	13.83	Unk-6	975		4		961	938
14	14.26	Unk-7	989		4		737	626
15	14.26	3-Pentanone	989	981	1	96-22-0	850	826
16	14.18	2-Pentanone	987	978	1	107-87-9	887	790
17	14.57	3,5-Dimethyloctane	999	-	3	15869-93-9	925	883
18	14.61	Decane	1000	1000	1	124-18-5	934	924
19	14.72	2,3,5,8-Tetramethyl-decane	1004	-	2	192823-15-7	842	810
20	14.91	4-Methyldecane	1012	1005	2	2847-72-5	909	850
21	15.09	Unk-8	1018		4		860	795
22	15.11	Unk-9	1019		4		927	898
23	15.61	1-Penten-3-one	1038	1029	1	1629-58-9	868	839
24	15.66	Unk-10	1040		4		870	768
25	15.59	(R)-α-Pinene	1037	1016	1	7785-70-8	944	940
26	15.82	Unk-11	1046		4		879	829
27	16.20	Unk-12	1060		4		804	772
28	16.31	Unk-13	1064		4		801	779
29	16.28	Toluene	1063	1041	1	108-88-3	927	923
30	16.41	Unk-14	1068		4		754	708
31	16.58	2-Propylheptanol	1074	-	3	10042-59-8	844	833
32	17.06	Unk-15	1092		4		824	783
33	17.47	Undecane	1107	1100	1	1120-21-4	904	887
34	17.46	Hexanal	1107	1099	1	66-25-1	918	916
35	17.78	Unk-16	1119		4		861	820
36	17.90	Unk-17	1124		4		824	821
37	18.17	Unk-18	1134		4		932	926
38	18.26	Unk-19	1137		4		722	686
39	18.25	Unk-20	1137		4		810	742
40	18.81	Ethylbenzene	1158	1138	1	100-41-4	937	904
41	19.05	o-Xylene	1167	1187	1	95-47-6	910	875

42	19.23	Unk-21	1174		4		802	722
43	19.38	Unk-22	1179		4		773	772
44	19.32	Unk-23	1177		4		925	910
45	19.39	Unk-24	1180		4		781	743
46	19.57	Unk-25	1187		4		755	742
47	20.31	Heptanal	1216	1196	1	111-71-7	897	883
48	20.29	Unk-26	1215		4		909	892
49	20.44	Unk-27	1221		4		940	831
50	20.56	1-Pentanol	1226	1248	1	71-41-0	871	803
51	20.67	Limonene	1230	1204	1	5989-54-8	943	933
52	20.78	(E)-2-Hexenal	1234	1225	1	6728-26-3	900	848
53	20.85	Unk-28	1237		4		745	733
54	21.01	Propylbenzene	1244	1219	2	103-65-1	898	789
55	21.05	1-Methoxy-2-propyl acetate	1245	1238	2	108-65-6	869	748
56	21.58	Unk-29	1254		4		801	746
57	21.82	Capryl chloride	1257	1260	2	111-85-3	840	799
58	21.94	Unk-30	1266		4		725	620
59	22.08	Unk-31	1276		4		726	714
60	22.10	Unk-32	1281		4		836	777
61	22.24	Hexyl acetate	1286	1290	1	142-92-7	894	889
62	22.37	Unk-33	1287		4		746	721
63	22.63	Tridecane	1293	1299	4	629-59-4	913	769
64	22.77	Octanal	1298	1302	4		910	757
65	22.79	1,2,3-Trimethylbenzene	1310	1339	1	620-14-4	906	853
66	23.02	Unk-34	1316		4		837	803
67	23.00	Unk-35	1318		4		810	808
68	23.14	Unk-36	1329		4		769	720
69	23.23	3-Hexenol acetate	1327	1337	1	3681-71-8	908	908
70	23.50	Unk-37	1334		4	2490-48-4	774	770
71	23.73	Farnesane	1339	1360	1	3891-98-3	872	825
72	23.76	1-Hexanol	1338	1356	1	111-27-3	889	876
73	24.02	Unk-38	1351		4		733	683
74	24.23	Unk-39	1362		4		820	753
75	24.48	(Z)-Hex-3-en-1-ol	1364	1394	1	928-96-1	941	937
76	24.65	Tetradecane	1376	1400	4	629-59-4	941	913
77	24.79	Unk-40	1386		4		808	745
78	24.91	Nonanal	1398	1404	4	124-19-6	924	923
79	24.94	β-Butoxyethanol	1407	1410	2	111-76-2	906	904
80	25.05	Unk-41	1414		4		913	840
81	25.27	Sorbaldehyde	1421	1441	2	142-83-6	902	887
82	25.39	Unk-42	1423		4		885	825
83	25.49	Unk-43	1428		4		919	860
84	25.60	Unk-44	1440		4		830	739
85	25.70	Unk-45	1446		4		801	754
86	25.88	Unk-46	1452		4		692	687
87	26.20	α-Cubebene	1458	1453	2	17699-14-8	887	847
88	26.23	Dipropylene glycol monomethyl ether	1463	1479	2	34590-94-8	848	742
89	26.19	Unk-47	1473		4		708	660

90	26.37	Unk-48	1473		4		751	722
91	26.32	(E,E)-2,4-Heptadienal	1485	1495	1	5910-85-0	882	874
92	26.30	2-Ethyl-1-hexanol	1491	1496	2	104-76-7	920	813
93	26.44	Pentadecane	1493	1500	2	544-76-3	872	703
94	26.49	Unk-49	1490		4		687	623
95	26.67	Unk-50	1500		4		661	559
96	26.80	Ylangene	1497	1491	2	14912-44-8	905	902
97	26.79	Unk-51	1496		4		773	758
98	27.03	Copaene	1504	1487	1	3856-25-5	918	916
99	27.27	Unk-52	1507		4		782	764
100	27.25	Unk-53	1518		4		694	677
101	27.48	Unk-54	1525		4		818	746
102	27.44	trans-α-Bergamotene	1524	1560	2	13474-59-4	909	873
103	27.46	6-Methyl-1-heptanol	1539	1524	2	1653-40-3	850	797
104	27.61	Benzaldehyde	1552	1530	1	100-52-7	942	940
105	27.70	Unk-55	1565		4		916	898
106	27.81	Unk-56	1563		4		901	883
107	27.77	Unk-57	1564		4		725	609
108	28.08	Unk-58	1574		4		896	826
109	28.35	Unk-59	1573		4		731	726
110	28.33	cis-α-Bergamotene	1579	1595	2	17699-05-7	951	932
111	28.47	Unk-60	1585		4		901	899
112	28.65	Unk-61	1583		4		758	735
113	28.72	Unk-62	1601		4		701	685
114	28.70	Unk-63	1620		4		913	903
115	28.82	Caryophyllene	1618	1599	2	87-44-5	915	915
116	29.02	1-Nonanol	1628	1658	1	143-08-8	921	891
117	29.08	Methyl benzoate	1640	1638	1	93-58-3	930	914
118	29.19	Unk-64	1646		4		777	773
119	29.24	Unk-65	1644		4		879	837
120	29.37	Unk-66	1652		4		856	752
121	29.32	Unk-67	1666		4		924	912
122	29.74	Unk-68	1670		4		833	753
123	29.87	Unk-69	1678		4		948	931
124	29.98	Unk-70	1682		4		938	933
125	30.15	Unk-71	1690		4		910	899
126	30.21	Unk-72	1687		4		762	752
127	30.42	Unk-73	1718		4		875	869
128	30.59	Unk-74	1737		4		925	904
129	30.77	Unk-75	1749		4		820	786
130	30.81	δ-Cadinene	1754	1744	2	483-76-1	921	905
131	31.01	Butyl dioxitol	1771	1781	2	112-34-5	907	892
132	31.22	Naphthalene	1771	1797	2	16728-99-7	833	810
133	31.25	Methyl salicylate	1784	1789	2	119-36-8	922	895
134	31.39	Unk-76	1798		4		700	695
135	31.57	Unk-77	1818		4		792	772
136	31.94	Unk-78	1837		4		844	810
137	31.97	Unk-79	1839		4		824	822
138	32.10	Benzyl Alcohol	1851	1861	1	100-51-6	947	946
139	32.22	Unk-80	1858		4		868	777

140	32.55	Phenylethyl Alcohol	1867	1872	1	60-12-8	899	873
141	32.76	Unk-81	1899		4		649	637
