# Supplementary figures and images for: Deciphering the Olive Fruit Volatilome: A Multivariate Approach to Assess Cultivar Variation and Biotic Stress Response in a Changing Agroclimatic Context
Source: Plants (Basel). 2026 Jul 22;15(14):2243. doi: 10.3390/plants15142243 (PMC13417002; doi:10.3390/plants15142243)

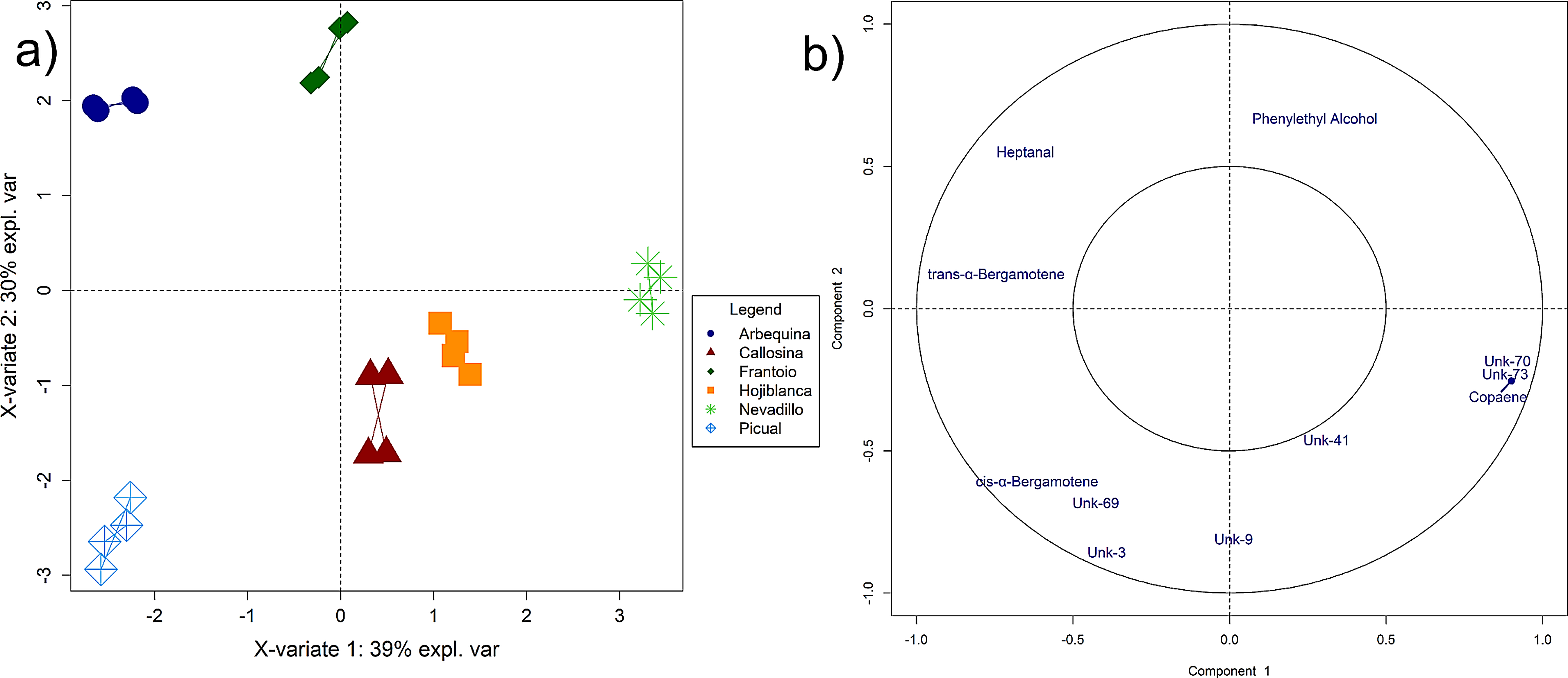

Supplement: Supplementary file 1 [file plants-15-02243-s001.zip › plants-4424238-supplementary/Figure_S1.png]
